# External Ophthalmomyiasis

**DOI:** 10.4269/ajtmh.26-0201

**Published:** 2026-07-09

**Authors:** Diana Verónica Guanochanga Vargas, Edy Quizhpe, Lucia Patricia Ceron Osejos, Emilie Tatiana Iglesias Vinocuña

**Affiliations:** ^1^ Hospital General del Sur de Quito, IESS, Quito, Ecuador;; ^2^ Facultad de Ciencias de la Salud “Eugenio Espejo”, Universidad UTE, Quito, Ecuador;; ^3^ Facultad de Ciencias Médicas, Universidad Central del Ecuador, Quito, Ecuador

A 14-year-old Afro-Ecuadorian girl from a rural district in Ecuador, Esmeraldas Province, presented with a pruritic lesion on the right upper eyelid that had been present for 1 week. Despite initial empirical beta-lactam therapy and anti-inflammatory treatment, there was progression of swelling along with new purulent discharge. The patient exhibited no systemic symptoms. Detailed right eye examination showed a palpable, mobile, nontender mass in the upper eyelid, which was responsible for mechanical ptosis without visual impairment and visual acuity 20/40 with manual elevation of the eyelid (Figure 1). A circular hyperpigmented lesion was observed on the tarsal conjunctiva. Initial diagnoses included stye and eyelid abscess. Imaging revealed a lobulated soft-tissue lesion with intralesional gas (Figure 2). Empirical intravenous beta-lactamase antibiotics were initiated.

**Figure 1.**
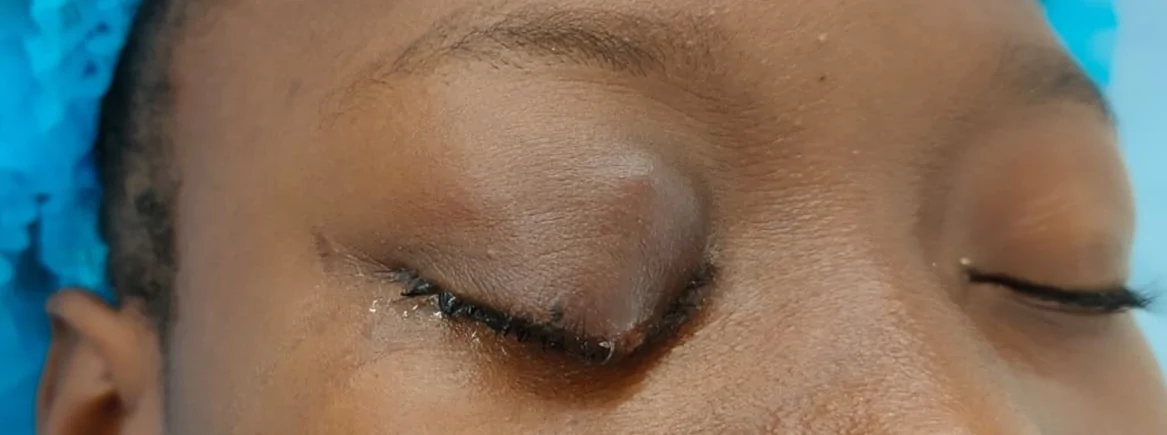
Right upper eyelid swelling with marked ptosis, accompanied by localized erythema and anterior eyelid inflammation.

**Figure 2.**
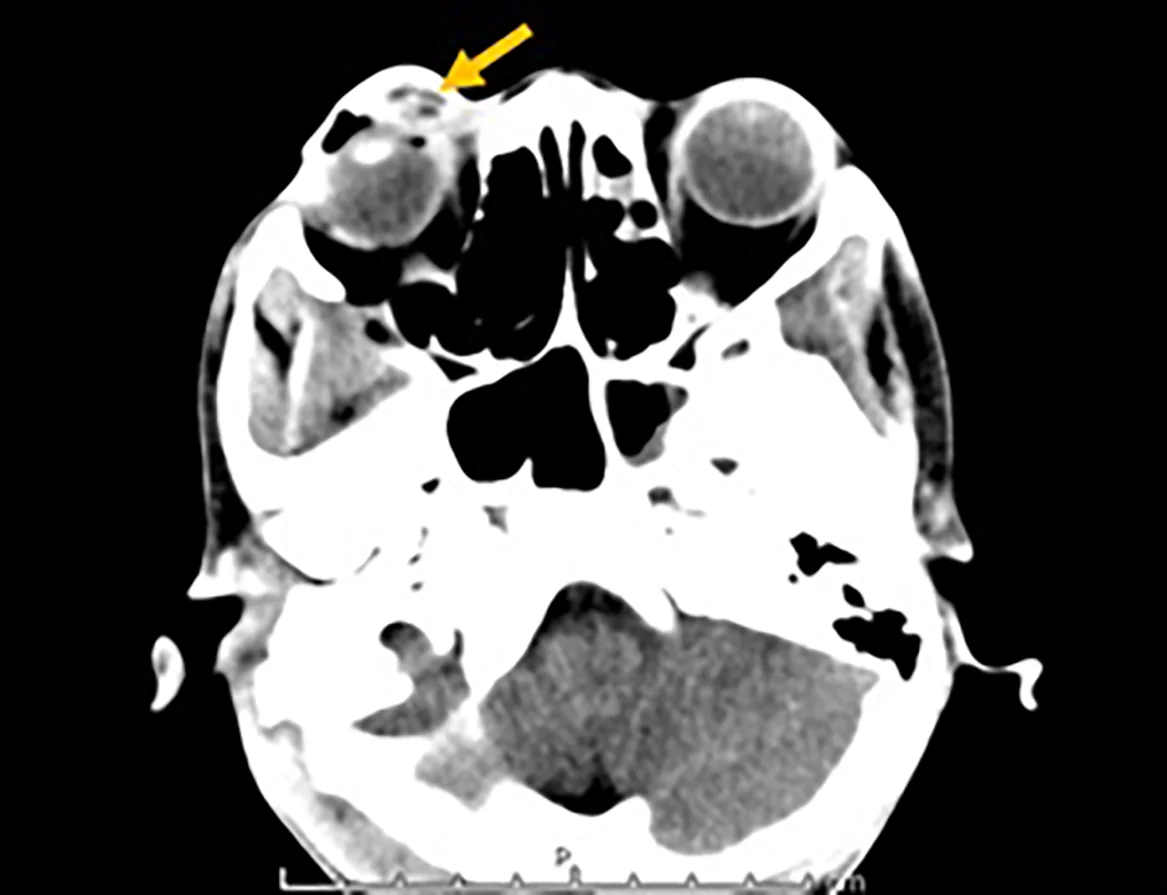
Axial orbital CT scan demonstrating right upper eyelid preseptal thickening and a lobulated soft-tissue lesion containing small intralesional gas foci (arrow), without evidence of intraorbital extension.

Eyelid eversion revealed a corneal ulcer (Figure 3) with a conjunctival protruding cystic structure suggestive of myiasis. A live larva measuring approximately 1 cm in length with numerous embedded black spines was removed (Figure 4). Histopathological examination confirmed myiasis; bacterial cultures were negative. The diagnosis was confirmed as palpebral cutaneous myiasis with superficial keratitis.

**Figure 3.**
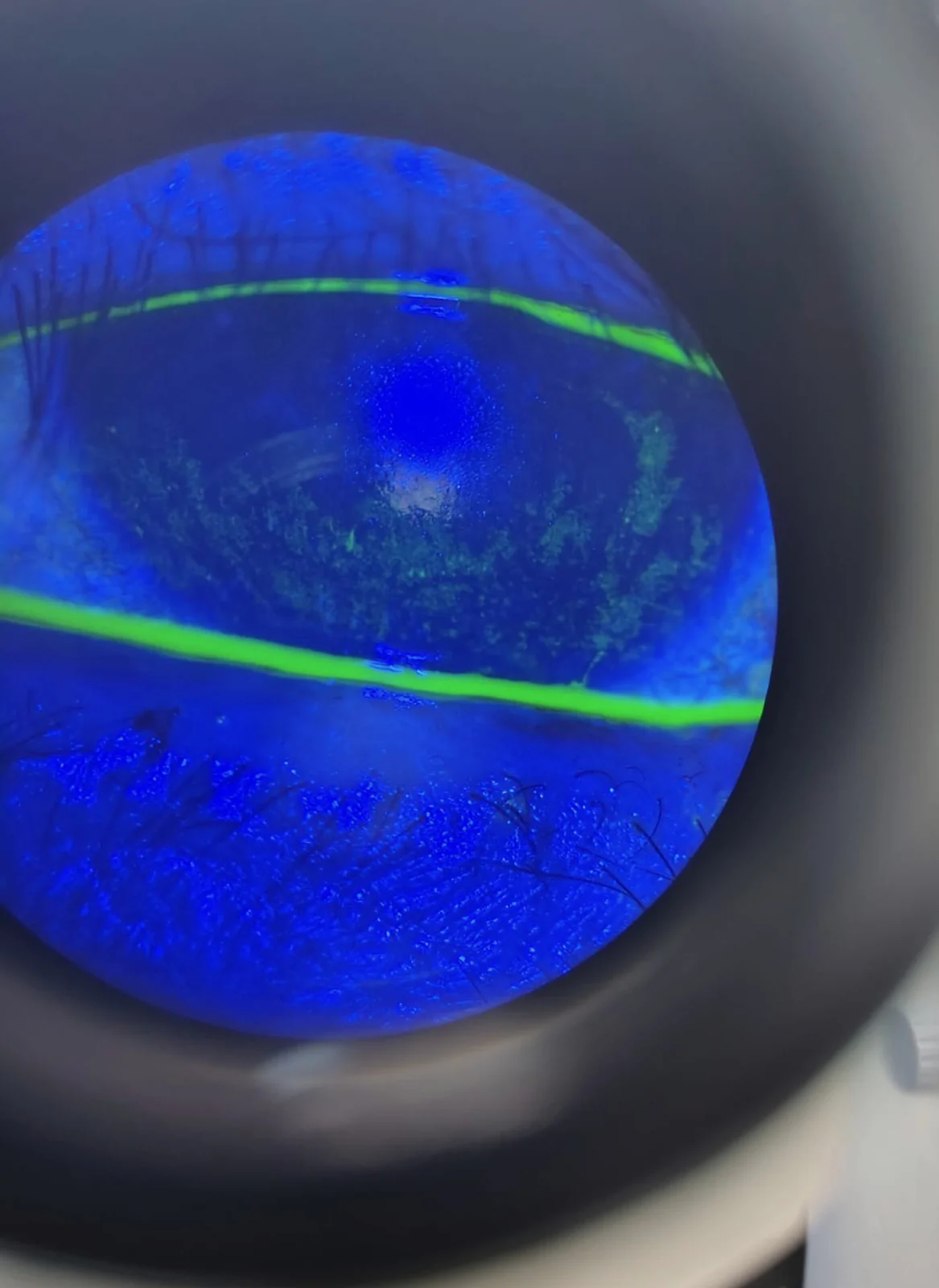
Slit-lamp examination with fluorescein staining under cobalt blue illumination, revealing diffuse punctate corneal epithelial staining and surface irregularity.

**Figure 4.**
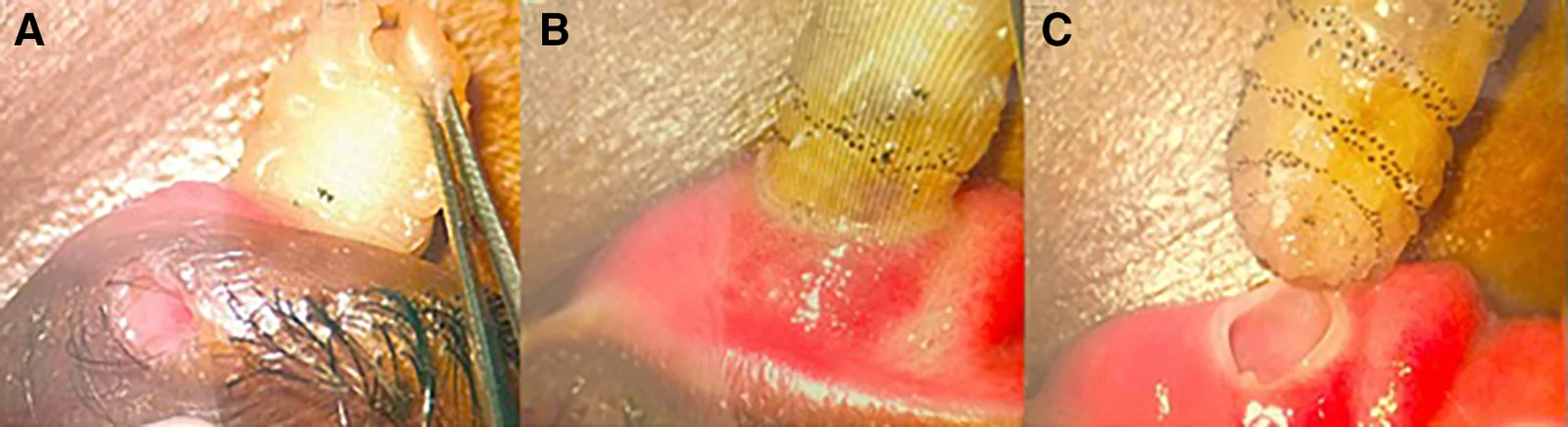
Right upper eyelid findings during evaluation and management: (**A**) conjunctival ulcer with a protruding larva; (**B**) larva partially extracted with forceps; (**C**) postextraction ulcer cavity showing exposed tissue.

Myiasis is a dipterous larval infestation common in tropical regions, including Ecuador.^1^ Ocular involvement is uncommon, which can result in delayed diagnosis.^2^^,^^3^ With orbital and palpebral involvement, if the larvae are not removed, complications may ensue.^4^^,^^5^ It is important to recognize this syndrome early to prevent ocular sequelae.^3^
